# Comparisons between myeloperoxidase, lactoferrin, calprotectin and lipocalin-2, as fecal biomarkers of intestinal inflammation in malnourished children

**DOI:** 10.15761/JTS.1000130

**Published:** 2016-03-25

**Authors:** Mara de Moura Gondim Prata, A Havt, DT Bolick, R Pinkerton, AAM Lima, RL Guerrant

**Affiliations:** 1 Department of Physiology and Pharmacology and INCT-Biomedicine, Faculty of Medicine, Federal University of Ceará, Fortaleza, Ceará, Brazil; 2 Center for Global Health, Division of Infectious Diseases and International Medicine, University of Virginia School of Medicine, Charlottesville, VA 22908, USA

**Keywords:** biomarkers, inflammation, malnutrition, enteropathy, myeloperoxidase, lactoferrin, calprotectin, lipocalin

## Abstract

Fecal biomarkers have emerged as important tools to assess intestinal inflammation and enteropathy. The aim of this study was to investigate the correlations between the fecal markers, myeloperoxidase (MPO), lactoferrin (FL), calprotectin (FC) and lipocalin-2 (Lcn-2), and to compare differences by breastfeeding status as well as normalization by fecal protein or by fecal weight. Simultaneous, quantitative MPO, FL, FC and Lcn-2, levels were determined in frozen fecal specimens collected from 78 children (mean age 15.2 ± 5.3 months) in a case-control study of childhood malnutrition in Brazil. The biomarker concentrations were measured by enzymelinked immunosorbent assay. The correlations among all biomarkers were significant (P<0.01). There were stronger correlations of fecal MPO with fecal lactoferrin and calprotectin, with lower, but still highly significant correlations of all 3 inflammatory biomarkers with Lcn-2 likely because the latter may also reflect enterocyte damage as well as neutrophil presence. Furthermore, the biomarker results with protein normalized compared to simple fecal weight normalized values showed only a slightly better correlation suggesting that the added cost and time for protein normalization added little to carefully measured fecal weights as denominators. In conclusion, fecal MPO correlates tightly with fecal lactoferrin and calprotectin irrespective of breastfeeding status and provides a common, available biomarker for comparison of human and animal model studies.

## Introduction

Fecal biomarkers have emerged as important tools to assess intestinal inflammation, whether due to inflammatory infections, such as shigellosis or *C. difficile* colitis, or to inflammatory bowel disease (IBD), be it ulcerative colitis or Crohn’s disease [1–4]. As relatively specific biomarkers of neutrophilic inflammation in the intestinal mucosal [5], these tests have the advantages of being noninvasive, rapid, simple and relatively inexpensive [6]. Several clinical studies have shown the usefulness of fecal biomarkers of inflammation in the diagnosis or in the tracking of disease activity; these include the stool measurement of sensitive biomarkers that include such neutrophil-granular proteins as lactoferrin (LF), myeloperoxidase (MPO), calprotectin (FC) and lipocalin-2 (Lcn-2). What is less clear are how these biomarkers compare with each other, especially with lower levels of inflammation seen with mild to moderate enteropathy in malnourished children, their relative dependence on breastfeeding status, and the necessity for protein normalization in their assessments. The purpose of this study was to address these key practical issues.

*Lactoferrin (LF)* is a long and widely used fecal biomarker of intestinal inflammation. It is an iron-biding glycoprotein present in secondary (specific) granules especially in mature neutrophilic granulocytes [1,7–9]. Although it provides an excellent quantifiable marker of neutrophilic inflammation, several exocrine cells also secrete lower amounts of this protein that are often present in lower concentrations in many fluids such as normal human milk, tears, synovial fluid, and serum. Its presence in breast milk has raised concerns about the validity of low levels of lactoferrin measured in the stools of exclusively or even partially breastfed children. LF is stable in fecal samples at room temperature for up to 5 days allowing samples to be sent to the laboratory [10]. During intestinal inflammation neutrophils infiltrate the mucosa and markedly increase LF levels that can be readily measured in feces or gut lavage fluid [6,11]. Studies evaluating lactoferrin in the diagnosis of IBD show that it exhibited similar performance to fecal calprotectin and correlated better than C-reactive protein with mucosal inflammation by endoscopy [12–14]. Joishy *et al.* [10] also found that FL correlated with disease activity indices and erythrocyte sedimentation rate in pediatric patients with IBD.

*Myeloperoxidase (MPO)* is a major component of the primary (azurophilic) granules especially in young neutrophils [15]. It is also present in much lower concentrations in monocytes and macrophages [16]. It is an enzyme that catalyzes hydrogen peroxide-mediated oxidation of halide ions to form such reactive substances as hypochlorous acid, tyrosyl radicals, and reactive nitrogen intermediates [15,17,18]. Although limited by its colorimetric bioassay in the past, the availability of specific antibody enables its use as a biomarker in humans as well as in animal models that reflects the range of neutrophil concentrations present in fecal contents with intestinal inflammation [16]. MPO has been observed both in the intestinal mucosa, and in gut lavage, and has the potential of monitoring disease activity and treatment outcomes in patients with ulcerative colitis [19].

*Calprotectin (FC)* is a calcium- and zinc-binding protein that inhibits metalloproteinase, has antifungal activity and induces apoptosis in cell culture [20,21]. It is the major protein found in monocytes, macrophages and constitutes 50–60% of neutrophil cytosolic proteins [22,23]. This protein is stable in stool samples kept at room temperature for up 7 days and resists heat and enzymatic degradation in the gut [24–26]. Although it is more variable in early infancy several studies have showed high FC levels are directly correlated with the quantification of the neutrophilic infiltrate in the gut mucosa as an indicator of infectious and inflammatory conditions [9,25,27–30].

*Lipocalin-2 (Lcn-2)* is a glycoprotein that is also known as neutrophil gelatinase-associated lipocalin (NGAL), siderocalin, uterocalin, or as a product of oncogene 24p3. It belongs to a family of small secreted glycoproteins (called lipocalins) [31]. This protein is expressed in several cells and, has been associated with primary and secondary granules of human neutrophils [31,32]. Expression of Lcn-2 is upregulated in tissue damaging conditions such as infection, kidney injury, cancer, ulcerative colitis and burn injury where production of free radicals occurs [33,34].

As many studies have used different fecal biomarkers of inflammation, we sought to determine how these fecal biomarkers compare with each other in a randomly chosen subset of fecal specimens from children in a study of malnutrition in Brazil. In this study we investigated fecal MPO as a biomarker of intestinal inflammation in comparison to FL, FC and Lcn-2. We also assessed whether breastfeeding status altered these relative biomarker associations. Furthermore, we examined the importance of using protein (*vs* weight) normalization of these biomarkers. We also derived correlations to relate quantified fecal lactoferrin to fecal MPO. This study was not designed to demonstrate how each biomarker correlates with nutrional/infection status, but rather to critically assess the performance and correlation of these important biomarkers in a set of samples from children in Northeast Brazil, an impoverished area for which these assays will be of critical importance in detecting childhood malnutrition.

## Methods

### Ethical considerations

The project was approved by the national IRB Conselho Nacional de Etica em Pesquisa (CONEP) at the Federal University of Ceara (UFC), Fortaleza, Ceara, Brazil; and the University of Virginia IRB for Health Services Research. All patients’ guardians were informed about the study goals and gave their written informed consent.

### Study design, population, and settings

This observational case-control study was carried out at the Federal University of Ceara and Center for Global Health of Virginia between August 2010 and September 2013. The study participants were identified through the Institute for the Promotion of Nutrition and Human Development (IPREDE). All children recruited to participate using the following inclusion criteria: between 6 and 26 months of age; the cases were children with malnutrition, defined by weight- for-age z-score (WAZ ≤ −2); the control group consisted of children who were recognized as nourished (WAZ ≥ −1) (without any specific illness). At enrollment, the parents answered a questionnaire regarding demographic, residence/location and breastfeeding status. The exclusion criteria included whether the children had critical health issues and/or who required extended hospitalization or if the caregiver or parents had a major cognitive deficit or were less than 16 years old [35].

### Stool collection

A stool sample was collected from each child into a sterile container. All samples were aliquoted and stored at −20°C in cryovials until sent to the Center for Global Health, University of Virginia for biomarkers analyses. We selected 78 of these children’s stool samples to represent high, medium and low MPO concentrations to compare a repeated quantitative MPO assay with normalization by either stool weight or measured protein concentrations simultaneously with lactoferrin, calprotectin and lipocalin-2 assays. This included 26 samples estimated to have low (n=26), medium (n=31), or high (n=21) MPO concentrations from initial studies done in Brazil.

### Inflammation biomarkers and protein measurements

Stool specimens were allowed to thaw at room temperature and were diluted 7-fold in buffer with protease inhibitors (RIPA, radioimmunoprecipitation assay buffer). The samples were vortexed, centrifuged and the supernatants were used to measure the biomarkers. The fecal myeloperoxidase (MPO), lactoferrin (FL), lipocalin-2 (Lcn-2) and calprotectin (FC) concentrations were measured using commercially available kits that employed polyclonal antibody-based enzyme-linked immunosorbent assay (ELISA) methods, according the manufacturer’s instructions. MPO and Lcn-2 levels were determined using human kits from R&D systems, Inc (Minneapolis, USA). The FL concentration was analyzed using a BD (IBD-SCAN); TechLab Inc. (Blacksburg, USA). The FC concentration was tested using a Calprotectin Elisa Assay Kit, Eagle Biosciences, Inc. (Nashua, USA). Total protein of each sample was assayed using the BCA Protein Assay Kit from Pierce (Pittsburgh, PA). The absorption was measured using an Epoch plate reader, Bio-tek Instruments, Inc. (USA). Units were expressed as ng/mg of stool or as ng/µg protein for protein normalized data.

### Data management and statistical analyses

Statistical analyses were performed using the SPSS statistical package version 21.0 (Chicago, USA). Independent- sample T-tests were used for comparison of variables between groups. A Pearson`s correlation was used when there were two quantitative variables. A *p* value of less than 0.05 was considered as statistically significant. The comparative analyses among the results of the four biomarkers were based on natural log.

## Results

### Study population, anthropometric, nutrition and biomarkers

The goal of this study was to investigate the correlation between fecal myeloperoxidase (MPO), lactoferrin (FL), calprotectin (FC) and lipocalin-2 (Lcn-2). Data showing how these fecal biomarkers correlate with nutritional/infection status are currently being analyzed separately as part of a much larger study. Table 1 shows the data from 78 children (42 females and 36 males) with an average age of 15.2 months (sd ± 5.3, range 6 to 26 months). In terms of infant feeding, 57 (73.1%) were being partially breastfed at the time of study enrollment, and 21 (26.9%) were fully weaned. All 4 biomarkers were evaluated at the same time after thawing for all 78 samples. The median and the range of concentration of the fecal markers were 8.42 ng/mg (1.75–32.9 ng/mg) MPO, 11.83 ng/mg (4.24–43.38 ng/mg) FL, 6.07 ng/mg (2.55–13.48 ng/mg) FC and 0.98 ng/mg (0.43–1.82 ng/mg) Lcn-2.

### Association between inflammatory biomarkers

**Figure 1:**
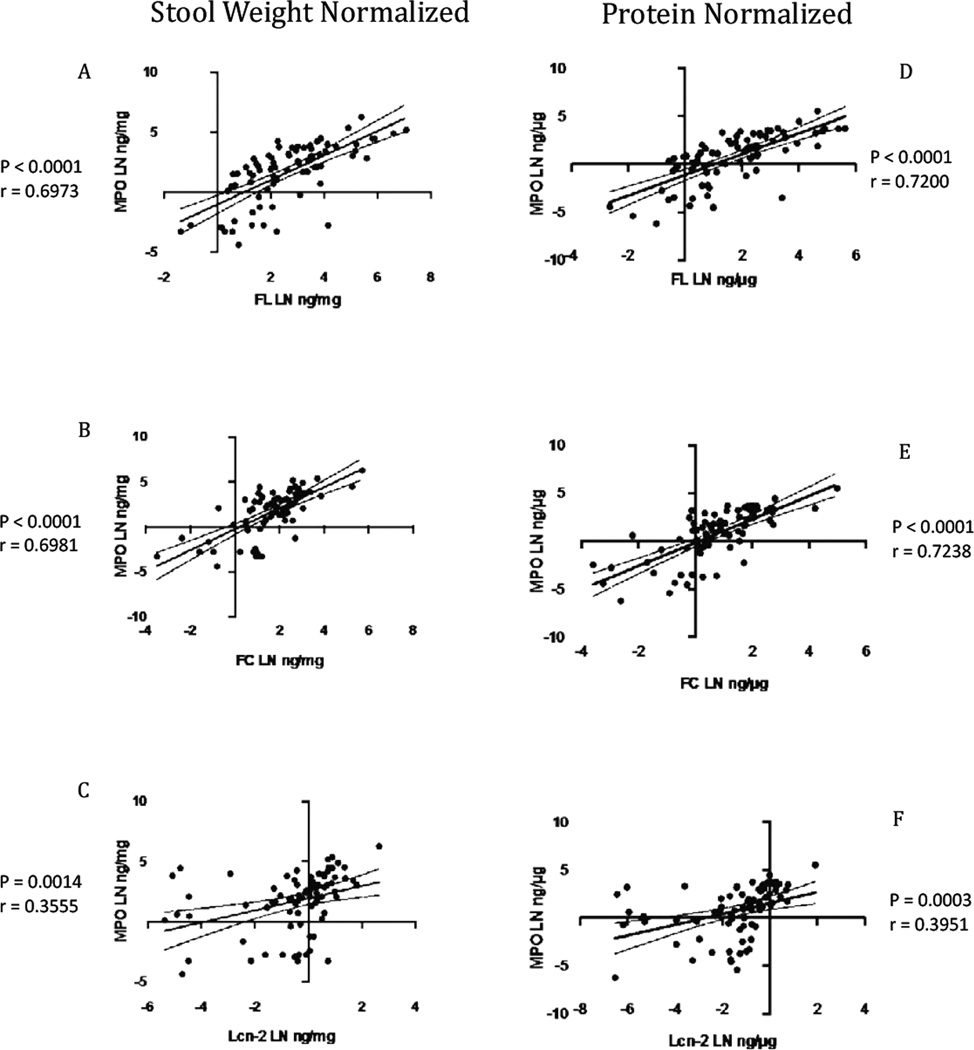
Pearson correlations of the natural log transformed (LN) between fecal MPO and the other biomarker with stool weight normalized and protein normalized in samples from malnutrition children. Stool weight normalized: (a) FL (P<0.0001; r=0.6973) (b) FC (P<0.0001; r=0.6981) (C) Lcn-2 (P=0.0014; r=0.3555). Protein normalized: (d) FL (P<0.0001; r=0.7200) (e) FC (P<0.001; r=0.7238) (f) Lcn-2 (P=0.0003; r=0.3951). MPO indicates fecal myeloperoxidase; FL, lactoferrin; FC, calprotectin; Lcn-2, lipocalin-2. The solid line represents the linear regression line, and the dashed curves denote the 95% confidence interval.

**Figure 2:**
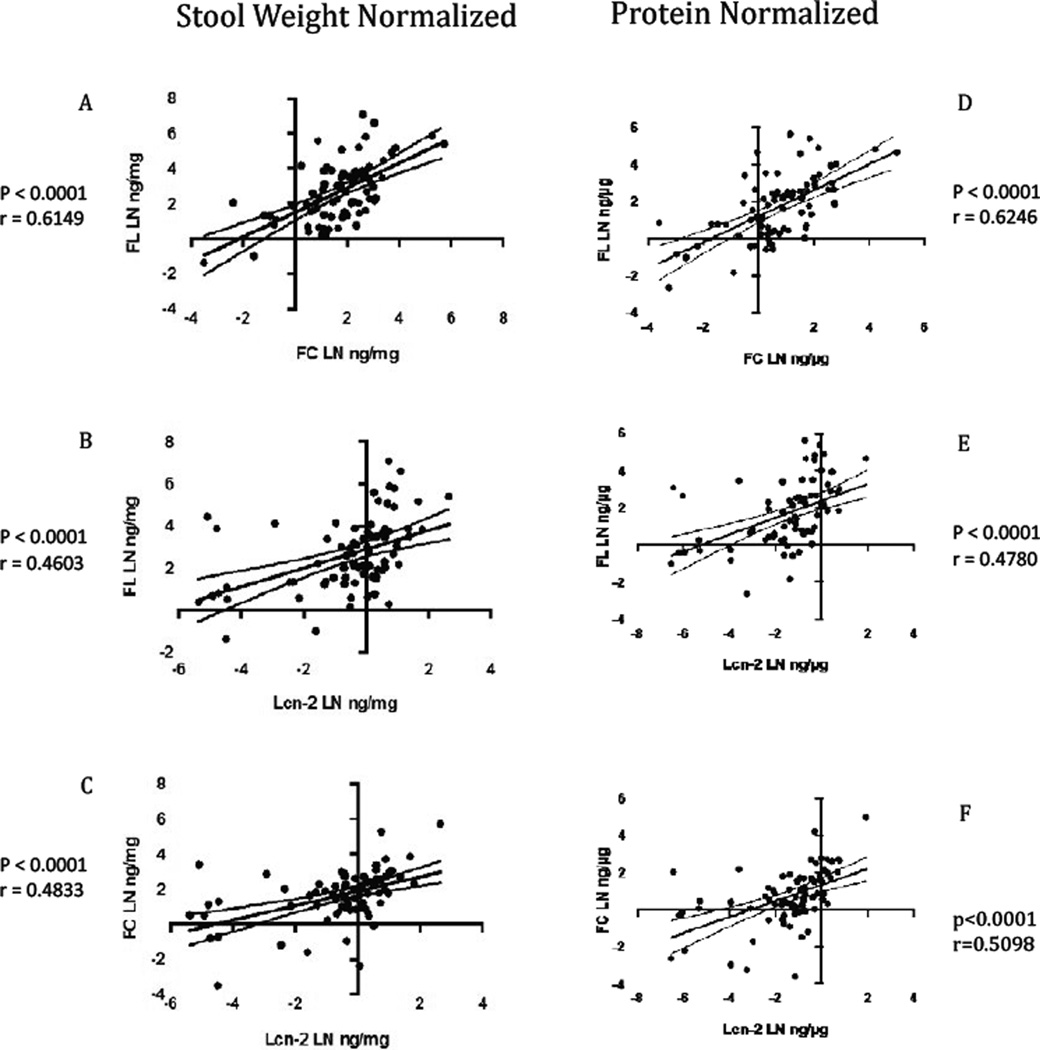
Pearson correlations of the natural log transformed (LN) between FL vs Lcn-2 and FC and Lcn-2 vs FC with stool weight normalized and protein normalized in samples from malnutrition children. Stool weight normalized: (a) FL vs FC (P<0.0001; r=0.6149) (b) FL vs Lcn-2 (P<0.0001; r=0.4603) (C) FC vs Lcn-2 (P<0.0001; R=0.4833). Protein normalized: (d) FL vs FC (P<0.0001; R=0.6246) (e) FL vs Lcn-2 (P<0.0001; R=0.4780) (f) Lcn-2 vs FC (P<0.0001; R=0.5098). MPO indicates fecal myeloperoxidase; FL, lactoferrin; FC, calprotectin; Lcn-2, lipocalin-2. The solid line represents the linear regression line, and the dashed curves denote the 95% confidence interval.

**Figure 3:**
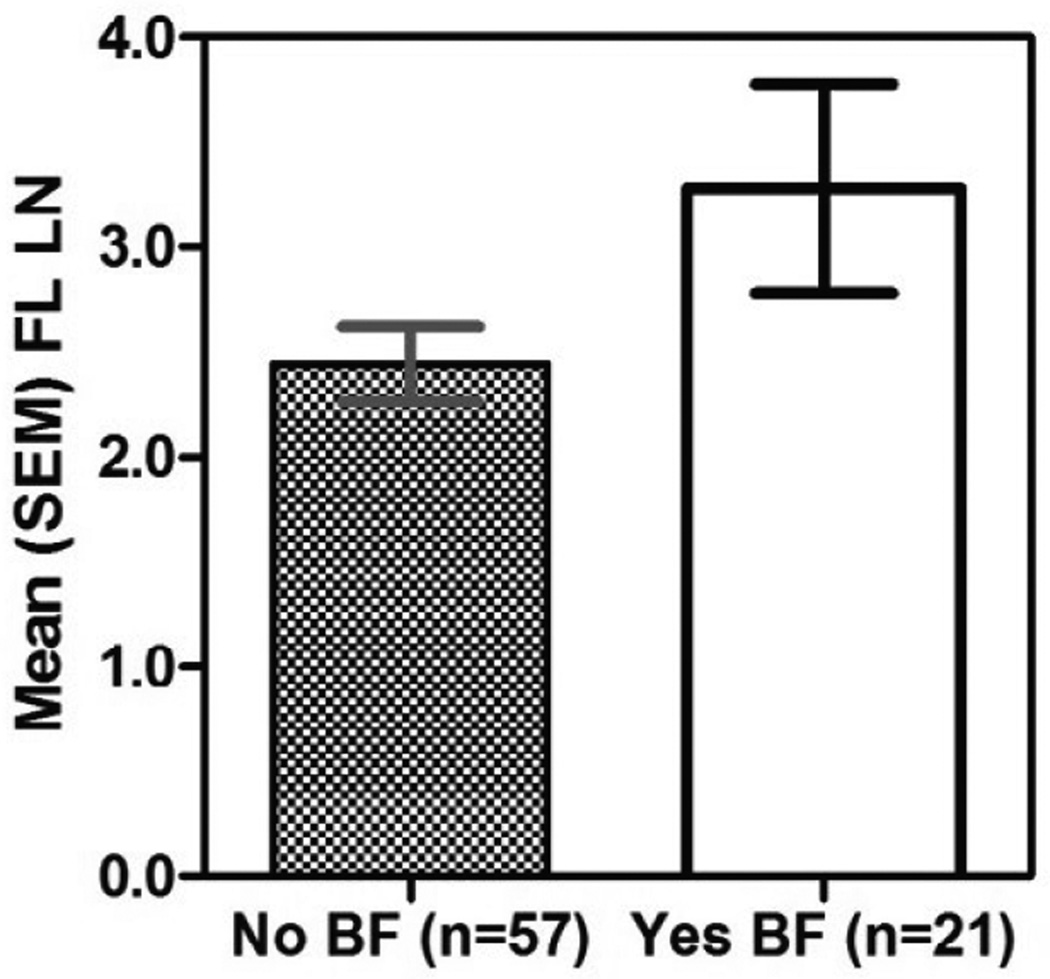
Graph of mean fecal lactoferrin (FL) based on breastfeeding status (p=0.051).

As shown in Figures 1 and 2 and in Table 1, all four biomarkers were highly significantly linearly correlated with one another. There were stronger correlations of fecal MPO with fecal lactoferrin and calprotectin, and lower, but still highly significant correlations of all 3 inflammatory biomarkers with lipocalin-2. Correlations with protein normalized compared with weight normalized results showed only slightly, but not significantly better correlations with protein normalized data. In addition, the correlations among the biomarkers with partially breastfed children were similar to those with nonbreastfed children (Figure 3). Additionally, we confirmed that sample collection date (a reflection of storage time) had no effect on results (Supplementary Figure 1 and Supplementary Table 1).

**Figure 4:**
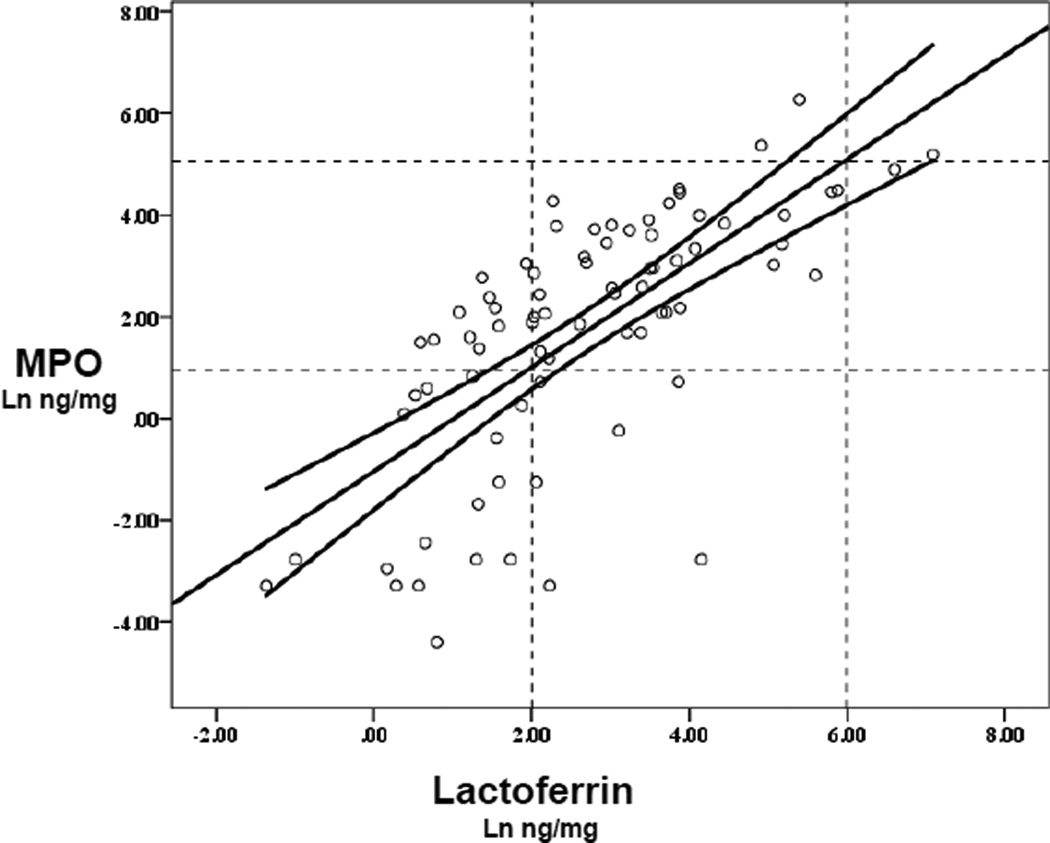
Correlation of Ln fecal lactoferrin with fecal myeloperoxidse (MPO) measurements, showing the linear regression line +s.e.mean (y = −1.04+1.02*x; n = 78; p<0.001; r=0.6973; R2=0.486). Dotted lines show published ranges of mild-moderate increases in lactoferrin of 7–400 ng/mg stool that intersect with the regression line at fecal MPO values of 2.8 and 169 ng/mg. The solid curves denote the 95% confidence interval.

In order to help relate findings in prior studies that used fecal lactoferrin to studies that use non-protein normalized MPO measurements in children or in animal model studies, we analyzed data showing the correlations of fecal lactoferrin with simultaneously run fecal MPO concentrations shown in Figure 4 (with non-protein normalized data as are typically used). Not shown are protein normalized data that are similar.

## Discussion

The need for simple, inexpensive, reliable, quantitative fecal biomarkers of intestinal inflammation is great. This study helps extend the feasibility of using these biomarkers from recognized inflammatory bowel diseases like ulcerative colitis and Crohn’s Disease (IBD) and invasive or inflammatory infections like shigellosis, salmonellosis, *Campylobacter* enteritis or *C. difficile* colitis to the increasingly troubling problem of children being evaluated for malnutrition and potential environmental enteropathy (EE) in early childhood in impoverished areas [36]. Given the huge magnitude of up to one third of the world’s poorest children who become stunting in their formative first 2 years of life (estimated 165 million children <5 years), early recognition of these children at greatest risk becomes urgently important as do any means of assessing innovative interventions [37]. Furthermore, biomarkers that are inexpensive, easily tested in or near field conditions and are not falsely altered by breast feeding are especially important as are correlations with animal models of enteric infections or enteropathy. Hence our focus in this report is on comparing available fecal biomarkers to assess the inflammatory component of potential EE in children living in an impoverished setting with varying degrees of under-nutrition. Data on how these biomarkers (as well as several additional measurements) correlate to nutrional/infection status are currently being analyzed as part of a much larger study. The results of these studies will be critical in validating the diagnostic and predictive efficacy as well as reproducibility of these fecal biomarkers.

We find excellent correlations among 3 recognized fecal biomarkers of intestinal inflammation. Especially helpful is the finding that partial breast feeding (in 57 of our 6–26 month old children) did not have significant effects on the relationships between these biomarkers, despite the recognized presence of low concentrations (relative to those found with inflammatory enteritis) of lactoferrin in breast milk and saliva and the recognized minor alterations of MPO by dietary protein intake [38]. Also very important and helpful is the minimal need for protein normalization of these fecal biomarkers, as this greatly alleviates the cost, time and materials needed for assay and helps support previous work done with simple volume or weight denominators [39].

In addition, we have developed a means to compare quantitative fecal lactoferrin and fecal MPO assay results in tests done at the same time on the same specimens. This enables us to evaluate risks and outcomes from studies done with these different assays at different times. Yet another advantage of fecal MPO measurements is that they can be directly compared with murine or other animal models of malnutrition and/or enteropathy and their pathogenesis.

An additional finding we note in these studies is that, while the 3 primary biomarkers of primary (azurophilic) or secondary (specific) neutrophil granules or of neutrophil cytosolic protein by MPO, lactoferrin or calprotectin respectively, correlate very well with each other, none correlate as well with fecal lipocalin, a marker that likely represents epithelial cell and tissue damage as well as the presence of neutrophils [40]. In studies Carlson and coworkers [41] showed no correlation between MPO and lipocalin in rectal segments in patients possibly indicating that these proteins are differentially assembled in inflamed intestinal mucosa. The expression Lcn-2 was reported to be promoted by production of chemokines that stimulate neutrophil-dependent NF-κB that was generated by IL-1β [42], therefore lipocalin likely correlates at least in part with epithelial damage. Indeed we have found that, in our murine models, fecal lipocalin may indeed be a better reflection of small bowel villous disruption that can be distinguished from neutrophilic inflammation per se (data not shown). However, these findings also raise the possiblilty that fecal lipocalin may well represent a complementary biomarker to assess intestinal epithelial damage in addition to intestinal inflammation.

In contrast to Lcn-2, both the levels of FL and FC correlated closely with MPO and each other. A number of studies suggest that FL and FC have comparable diagnostic accuracy for more severe inflammatory bowel disease [43]. Joishy and coworkers [10] demonstrated that the combination of FC and FL may further increased the sensitivity and specificity of these tests in predicting intestinal inflammation. A prospective study in patients with Crohn’s disease showed a significant correlation between calprotectin and lactoferrin levels (r=0.62, p=0.003) [44] that is similar to our findings with either weight or protein normalized results.

Previous reports have suggested that fecal lactoferrin in children and young adults have an upper limit of normal for healthy control individuals of 7.25 mg/ml [45], while another study conducted in healthy adult volunteers by Guerrant *et al.* [46] suggested that mild-moderate inflammation was <400 ng/mg; and high LF was ≥ 400 ng/mg (or /ul) as seen in 10 of 12 volunteers who developed classical febrile, inflammatory diarrhea with experimental shigellosis. Hence, we noted these ranges in Figure 3 for comparison of our fecal lactoferrin and MPO measurements (by stool weight) in order to compare the original fecal lactoferrin measurements in healthy children and in volunteers after experimental *Shigella* infections [45,46]. Although some have raised concerns about the amount of a protein or biomarker itself being present in milk seen with breastfeeding [47], we did not find significant differences in fecal biomarker correlations with or without breastfeeding.

Finally, of considerable practical importance regarding assay cost and time, although there were consistently slightly better correlations with protein normalized biomarker data in these fecal specimens, these effects were minimal and not statistically significant when compared with results just based on measured weight, thus the extra time and expense of also assaying fecal protein concentrations is not critically necessary.

In conclusion, currently available fecal biomarkers, MPO (a primary granule marker), lactoferrin (a secondary granule marker) and calprotectin (a neutrophil cytoplasmic marker) can provide simple, non-invasive assessments of intestinal inflammation that correlate closely with each other, do not require protein normalization and show similar correlations independently of breastfeeding status. Furthermore, MPO can also be used to compare results in animal models with human studies. In addition, although lipocalin-2 correlates less tightly with the neutrophil markers, it may provide a complementary marker of epithelial cell disruption or damage.

## Supplementary Material

Supplemental Data

## Figures and Tables

**Table 1:** Baseline characteristics of study population (n=78).

Characteristic	Description
Age in months (Mean, sem)	15.2 (5.3)
Sex (n, %)	
Male	42 (53.8)
Female	36 (46.2)
Breastfeeding (n, %)	
yes	57 (73.1)
no	21 (26.9)
MPO (ng/mg) median (25th, 75th percentile)	8.42 (1.75 – 32.97)
Lcn-2 (ng/mg) median (25th, 75th percentile)	0.98 (0.43 – 1.82)
FC (ng/mg) median (25th, 75th percentile)	6.07 (2.55 – 13.48)
FL (ng/mg) median (25th, 75th percentile)	11.83 (4.24– 43.38)

MPO=Myeloperoxidase Lcn-2=Lipocalin-2 FC= fecal calprotectin FL= fecal lactoferrin

**Table 2:** Pearson correlations (*r* values) for inflammatory Biomarkers using stool weight or protein normalized values.

Correlation Inflammatory Biomarkers		Stool Weight Normalized	Protein normalized
MPO	FL	0.6973	0.7200
	FC	0.6981	0.7238
	Lcn-2	0.3555*	0.3949*
FL	FC	0.6149	0.6246
	Lcn-2	0.4603	0.4780
FC	Lcn-2	0.4833	0.5098

* Correlation is significant P<0.01 [P=0.001]l(2-tailed). All other correlations are significant at P<0.001, Correlations are similar with breastfeeding. MPO indicates myeloperoxidase; Lcn-2, lipocalin-2; FC, fecal calprotectin; FL, fecal lactoferrin.

## Abbreviations

MPO: myeloperoxidase
FL: fecal lactoferrin
FC: fecal calprotectin
Lcn-2: lipocalin-2
EE: environmental enteropathy
IBD: inflammatory bowel disease
