# Supplementary material for: Comparisons between myeloperoxidase, lactoferrin, calprotectin and lipocalin-2, as fecal biomarkers of intestinal inflammation in malnourished children
Source: J Transl Sci. Author manuscript; Available in PMC 2016 Oct 12. (PMC5061054; doi:10.15761/JTS.1000130)
Supplement: Supplemental Data [file NIHMS812475-supplement-Supplemental_Data.docx]

Supplemental Figure 1


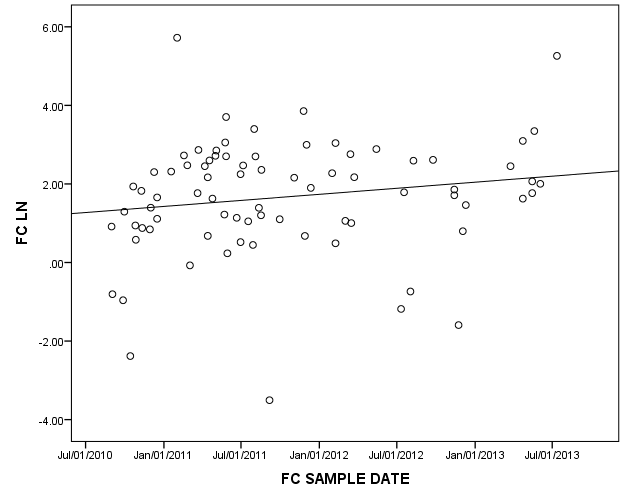

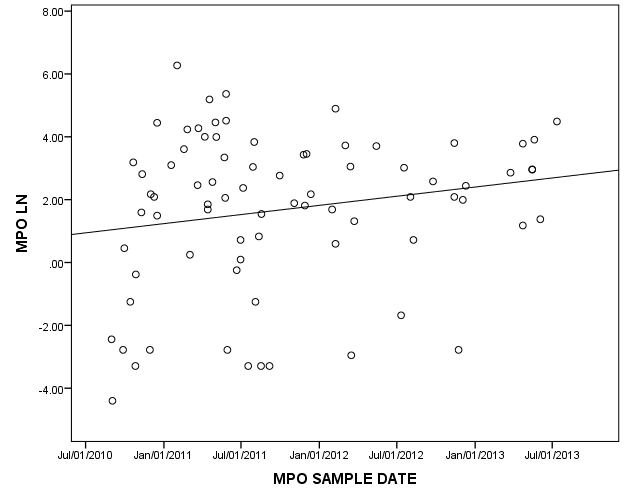

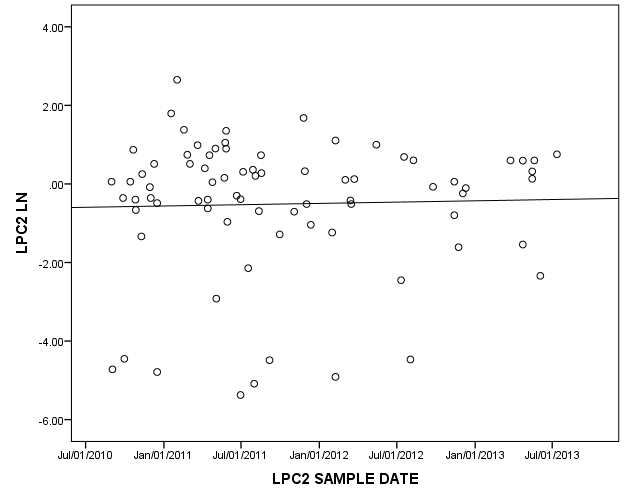


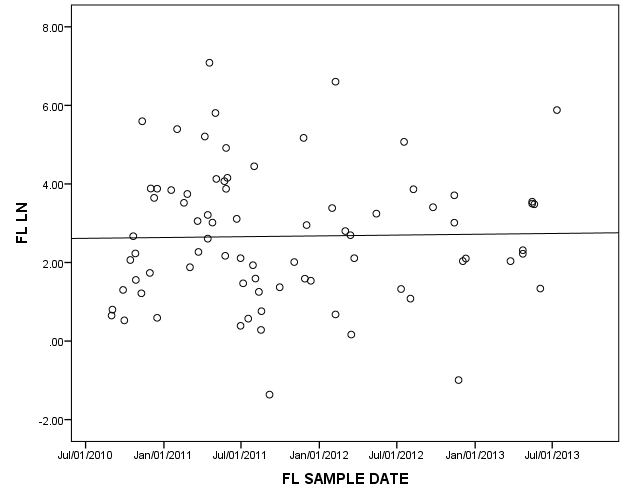


| **Correlations** | | | | | |
| --- | --- | --- | --- | --- | --- |
|  | | MPOngmg_LN | LPC2NLngmg | CPTNLngmg | LFFLNngmg |
| SAMPLE DATE | Pearson Correlation | .190 | .031 | .169 | .020 |
|  | Sig. (2-tailed) | .095 | .790 | .139 | .864 |
|  | N | 78 | 78 | 78 | 78 |

Supplemental Figure 2. Correlations of fecal biomarkers myeloperoxidase (MPO), fecal calprotectin (FC), lipocalin-2 (LPC2), and fecal lactoferrin (FL) based on collection date.
